# Wild-Type MIC Distribution for Re-evaluating the Critical Concentration of Anti-TB Drugs and Pharmacodynamics Among Tuberculosis Patients From South India

**DOI:** 10.3389/fmicb.2020.01182

**Published:** 2020-06-30

**Authors:** Azger Dusthackeer, Shainaba A. Saadhali, Manonanthini Thangam, Sameer Hassan, Mahizhaveni Balasubramanian, Angayarkani Balasubramanian, Geetha Ramachandran, A. K. Hemanth Kumar, Kannan Thiruvenkadam, Govindarajan Shanmugam, Christy Rosaline Nirmal, Sam Ebenezer Rajadas, Sucharitha Kannappan Mohanvel, Rajesh Mondal

**Affiliations:** ^1^Department of Bacteriology, National Institute for Research in Tuberculosis, Chennai, India; ^2^AU-KBC Research Centre, Anna University, Chennai, India; ^3^Division of Neurogeriatrics, Karolinska Institutet, Solna, Sweden; ^4^Department of Biochemistry, National Institute for Research in Tuberculosis, Chennai, India; ^5^Department of Epidemiology, National Institute for Research in Tuberculosis, Chennai, India; ^6^CAS in Botany, University of Madras, Chennai, India

**Keywords:** tuberculosis, minimum inhibitory concentration (MIC), critical concentration, drug resistance, pharmacodynamics (PD)

## Abstract

The World Health Organization (WHO) has developed specific guidelines for critical concentrations (CCs) of antibiotics used for tuberculosis (TB) treatment, which is universally followed for drug susceptibility testing (DST) of clinical specimens. However, the CC of drugs can differ significantly among the mycobacterial species based on the population, geographic location, and the prevalence of the infecting strain in a particular area. The association between CC and the minimal inhibitory concentration (MIC) of anti-TB drugs is poorly understood. In this study, we assessed the MICs of anti-TB drugs, including isoniazid (INH), rifampicin (RMP), moxifloxacin (MXF), ethambutol (ETH), and *p*-aminosalicylic acid (PAS) on drug-sensitive Mtb isolates from pulmonary TB patients in South India. The MIC assays performed using solid- and liquid-growth media showed changes in the CC of a few of the tested antibiotics compared with the WHO-recommended levels. Our observation suggests that the WHO guidelines could potentially lead to overdiagnosis of drug-resistant cases, which can result in inappropriate therapeutic decisions. To evaluate the correlation between drug-resistance and CC, we performed the whole-genome sequencing for 16 mycobacterial isolates, including two wild-type and 14 resistant isolates. Our results showed that two of the isolates belonged to the W-Beijing lineage, while the rest were of the East-African–Indian type. We identified a total of 74 mutations, including five novel mutations, which are known to be associated with resistance to anti-TB drugs in these isolates. In our previous study, we determined the serum levels of INH and RMP among the same patients recruited in the current study and estimated the MICs of the corresponding infected isolates in these cases. Using these data and the CCs for INH and RMP from the present study, we performed pharmacodynamics (PD) evaluation. The results show that the PD of RMP was subtherapeutic. Together, these observations emphasize the need for optimizing the drug dosage based on the PD of large-scale studies conducted in different geographical settings.

## Introduction

Tuberculosis (TB) is an infectious disease caused by *Mycobacterium tuberculosis*. Globally, it is the leading cause of mortality from a single infectious disease agent (World Health Organization Technical Report, 2018). The World Health Organization has developed an “End TB” strategy to strengthen global TB control and to improve the treatment outcome (World Health Organization, 2018). It is anticipated that the TB epidemic will be significantly curtailed in the next 20 years, in countries imposing the End TB Strategy (World Health Organization, 2018). The current strategy of the TB treatment relies on drug susceptibility testing (DST) to identify patients with drug-resistant tuberculosis (Kim, 2005). Traditionally, the DST for *M. tuberculosis* complex (MTBC) organisms involves analyzing a single critical concentration (CC) of the drug, which provides information on the drug-susceptibility of MTBC to each of the anti-TB agent (Coeck et al., 2016; Ruesen et al., 2018). The CC is defined as the lowest concentration of an anti-TB drug that can inhibit the growth of at least 95% of wild-type *M. tuberculosis in vitro*, in solid media (Lowenstein–Jensen; LJ, slopes) or liquid medium (mycobacteria growth indicator tubes; MGIT) (Werngren et al., 2012). According to the WHO guidelines, the CCs for the first-line anti-TB drugs in LJ media and MGIT, respectively, are as follows: isoniazid (INH; 0.2 and 1.0 mg), rifampicin (RMP; 40 and 1.0 mg), ethambutol (EMB; 2.0 and 5.0 mg), and pyrazinamide (100 mg/L only in MGIT) (Canetti et al., 1963; World Health Organization, 2012). The WHO-recommended CCs of anti-TB drugs are universally followed to determine the susceptibility pattern of MTBC in DST.

However, growing evidence suggests that there are differences in CCs among various populations depending on the geographical location and the type of MTBC isolates prevalent in the community (Pasipanodya et al., 2012). Furthermore, the genotype and the polymorphism of the infecting MTBC can also affect the CC of anti-TB drugs (Leandro et al., 2013). Thus, while the WHO-recommended CCs have been followed uniformly for some anti-TB agents, other drugs have been modified from this international standard to accommodate the diversity in anti-TB drugs (Somasundaram and Paramasivan, 2006). Besides, the relevance of the WHO-recommended CC of drugs on the susceptibility of divergent MTBCs prevalent in a particular geographical location, as well as the clinical outcome of patients with TB, remains uncertain. The global pandemic of TB not only affects people of different genetic backgrounds but is also highly inconsistent and shows variable pharmacokinetics (PK) of anti-TB drugs among the population (Gumbo, 2010).

Because India is a densely populated country with significant diversity in cultural background, food habits, and socioeconomic status, the outcome of anti-TB treatment at the population level is expected to be heterogeneous (Naidoo et al., 2017). Hence, variability in the MICs of MTBC isolates and the prevalence of different isolates within communities are inevitable. This variability can also contribute to the variable rates of PK/PD and the area under the curve (AUC) concentration in patient samples treated with anti-TB drugs (Naidoo et al., 2017).

In this study, we assessed the CC of anti-TB drugs in wild-type MTBC isolates that are prevalent among pulmonary TB patients of South India. We used both solid and liquid media to determine the CC of MTBC isolates and correlated it with the serum PD values. Finally, we performed whole-genome sequencing of the selected drug-sensitive and drug-resistant MTBC isolates and correlated the data with the MIC/CC results. Our findings show a significant variation in the CC of MTBC isolates in clinical specimens, which may contribute to the overestimation of drug-resistant cases compared with the WHO standards. The practical outcome of this study may provide new insight into the WHO criteria for the CC of first-line anti-TB drugs.

## Materials and Methods

### Bacterial Isolates

Wild-type clinical isolates of *M. tuberculosis* were isolated from the sputum samples of patients admitted for the PK study [A study to determine factors that influence plasma concentrations of first-line anti-TB drugs in adult TB patients receiving treatment according to Revised National Tuberculosis Control Program (RNTCP, India) guidelines]. The isolates were grown in Lowenstein–Jenson (LJ) medium. A set of six well-characterized, drug-resistant isolates was used as controls. Ethical approval for the study was obtained from the Institutional Ethics Committee, National Institute for Research in Tuberculosis.

Inclusion and exclusion criteria were as follows: MTBC cultures isolated from patients' specimen before the initiation of anti-TB treatment were used for the study. Those isolates from the patient who had received the treatment were not used for the study.

### Statistical Analysis and Sample Size Calculation

All data in the questionnaire were entered into a Microsoft Excel sheet and analyzed using the STATA version 15.1 (StataCorp, Texas, USA). The data were cross-tabulated using frequency and percentage. The principal component analysis was performed to identify the relatedness between the drugs MIC values and DST patterns.

The comparative study design was adopted to perform a more efficient investigation assuming 5% marginal error, 95% confidence interval, and a 5% chance of contamination, and the other possibility of missing in the 900 possible specimens collected for the PK study (Ramachandran et al., 2017). The proportion of wild-type isolates was kept as half (50%); with this, the study sample size was 284.

### MIC Determination

The required antibiotics were procured from Sigma-Aldrich (St. Louis, MO, USA). The MIC determination was carried out by performing three methods parallelly following the methods described elsewhere (Schon et al., 2009; Jureen et al., 2010). The INH stock solution was prepared with double-distilled water. RMP was dissolved with dimethylformamide, and further dilutions were made using double-distilled water. Middlebrook 7H11 (7H11) agar containing oleic acid albumin dextrose catalase (OADC) plates with different concentrations of drugs were prepared individually by mixing the required concentration of the drug with 7H11 just before the pouring the plate. The isolates were subcultured in the LJ medium for 3 weeks before the start of the experiment. For the experiment, a smooth suspension of *M. tuberculosis* colony was prepared in Middlebrook 7H9 (7H9) broth containing OADC and left undisturbed for 5 min. The cell supernatant was matched to 0.5 McFarland Standard with 7H9. Of the resulting suspension, 5 μl was spotted on the drug-containing 7H11 plates in triplicate. A 1:100 dilution of the cells was further made and spotted on the drug-free 7H11 plate, which served as a control. The estimation of MIC for the drugs INH and RMP was performed at 0.008, 0.04, 0.2, 1, and 5 μg/ml and 4, 8, 16, 32, 64, and 128 μg/ml concentration, respectively, in 7H11 agar plates. The plates were incubated at 37°C until growth appears. The growth in 1:100 dilutions was assured in drug-free 7H11 while reading the results for each isolate. The lowest concentration of the drug with no growth or more than 99% inhibition in 7H11 was considered as MIC (Schon et al., 2009). Similarly, the assay was done in MGIT 960 (liquid culture) following the manufacturer's protocol. The concentrations of both the drugs ranged between 0.015 and 512 μg/ml. MYCOTBI (Thermo Fischer Scientific, Massachusetts, USA) was used to determine the DST according to the manufacturer's protocol. In this method, the following are the concentration ranges for each of the drugs: INH (0.03, 0.06, 0.125, 0.25, 0.5, 1, 2, 4 μg/ml), RMP (0.125, 0.25, 0.5, 1, 2, 4, 8, 16 μg/ml), ofloxacin (OFX) (0.125, 0.25, 0.5, 1, 2, 4, 8, 16 μg/ml), moxifloxacin (MXF) (0.06, 0.125, 0.25, 0.5, 1, 2, 4, 8 μg/ml), amikacin (AMI) (0.125, 0.25, 0.5, 1, 2, 4, 8, 16 μg/ml), streptomycin (STR) (0.25, 0.5, 1, 2, 4, 8, 16, 32 μg/ml), rifabutin (RFB) (0.125, 0.25, 0.5, 1, 2, 4, 8, 16 μg/ml), para-aminosalicylic acid (PAS) (0.5, 1, 2, 4, 8, 16, 32, 64 μg/ml), ethionamide (ETM) (0.3, 0.6, 1.2, 2.5, 5, 10, 20, 40 μg/ml), cycloserine (CYC) (2, 4, 8, 16, 32, 64, 128, 256 μg/ml), kanamycin (KAN) (0.6, 1.2, 2.5, 5, 10, 20, 40 μg/ml), and ethambutol (ETH) (0.5, 1, 2, 4, 8, 16, 32 μg/ml). At least five of the isolates were repeated and *M. tuberculosis* H37Rv was used multiple times in each method, and six isolates from the panel of cultures for proficiency testing were used in all three types of methods.

### PK/PD Indices

The PK variability, including maximum peak concentration (*C*_max_), exposure, or area under the time–concentration curve (AUC), was obtained using the plasma drug levels, measured at 2, 4, 6, and 8 h time points, from TB patients in Chennai, South India. Data for 100 patients are available in our institute (Hemanth Kumar et al., 2016). Pharmacodynamic (PD) indices such as 24-h AUC/MIC ratio and the peak/MIC ratio were calculated with the CC determined in this study using the MGIT 960 method.

### *In silico* Analysis of the Whole-Genome Sequence Data

Sixteen isolates (2 sensitive and 14 resistant) were sequenced using paired-end Illumina MiSeq (NCBI Submission ID: SUB6703345). The quality of the sequence reads was analyzed using the FASTQC tool (McKenna et al., 2010). The sequence length of all the sequence reads was uniformly 150 bp. The reads with the quality Phred score, <28, were treated as lower quality and were trimmed off using a Trimmomatic tool (Bolger et al., 2014). Subsequently, the filtered sequence reads with better quality were aligned to the reference genome H37Rv using the short-read aligner Bowtie2 (Langmead and Salzberg, 2012). The aligned files were indexed and sorted using the SAMtools (an alignment file manipulating tools) (Li et al., 2009). The PCR duplicate in SAM files was removed and converted to BAM files using SAM tools to reduce the computational complexity of the analysis. The BAM files were analyzed for variants using mpileup in SAMtools followed by VarScan2, and in another way, the same BAM files were analyzed using Free Bayes and Genome Analysis Tool Kit (GATK) to generate VCF files (McKenna et al., 2010; Garrison and Marth, 2012; Koboldt et al., 2013). The single-nucleotide polymorphisms (SNPs) predicted from the variant analysis tools were compared, and only common SNPs with depth (DP) more significant than 10 and minimum mapping quality as 30 were filtered using VCF tools. Furthermore, the SNPs were annotated using SnpEff (Cingolani et al., 2012) to obtain the effect and location of variants. Using SNP Relate, an R/Bioconductor package (Zheng et al., 2012), principal component analysis (PCA) was made from a multisample variant file to study the variance among the samples. A small set of 12 PCA-correlated SNPs were identified, which are associated with the variation among samples. SNPs present in the wild-type samples NIRT_2 and NIRT_3 were compared with other drug-resistant samples NIRT_1 and NIRT_4 to NIRT_16. The variants in the multisample file were further clustered using the standard algorithm K-means clustering, and a dendrogram was made to study the phylogenetic relationship among samples. Each sample was also analyzed to find the strain type and the associated genotypes of *M. tuberculosis*. The most common genotypes among the genotypes of the same strain type were removed using customized Python script, and the resulting most significant variants were further analyzed and annotated to study the variant effects using tbvar, KVarQ, TBProfiler, and PhyResSE (Joshi et al., 2014; Steiner et al., 2014; Coll et al., 2015; Feuerriegel et al., 2015).

## Results

**Figure 1:**
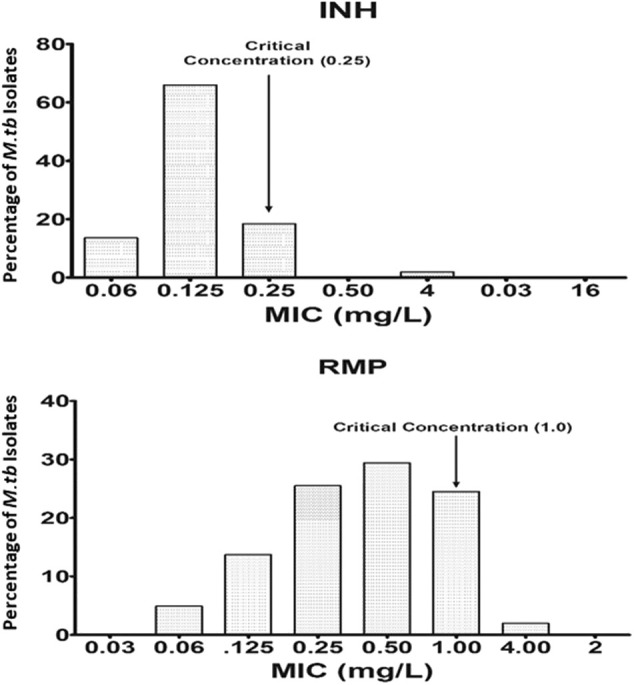
Wild-type minimal inhibitory concentration (MIC) distribution of isoniazid (INH), and rifampicin (RMP) in 276 isolates of *M. tuberculosis* performed in 7H11 agar plates (solid media). The critical concentration determined in this study is indicated by the arrow and is the same as recommended by WHO using 7H10 agar plates. *Y*-axis represents the percentage of *M. tuberculosis* isolates, and the *X*-axis represents the MIC of the corresponding drugs.

**Figure 2:**
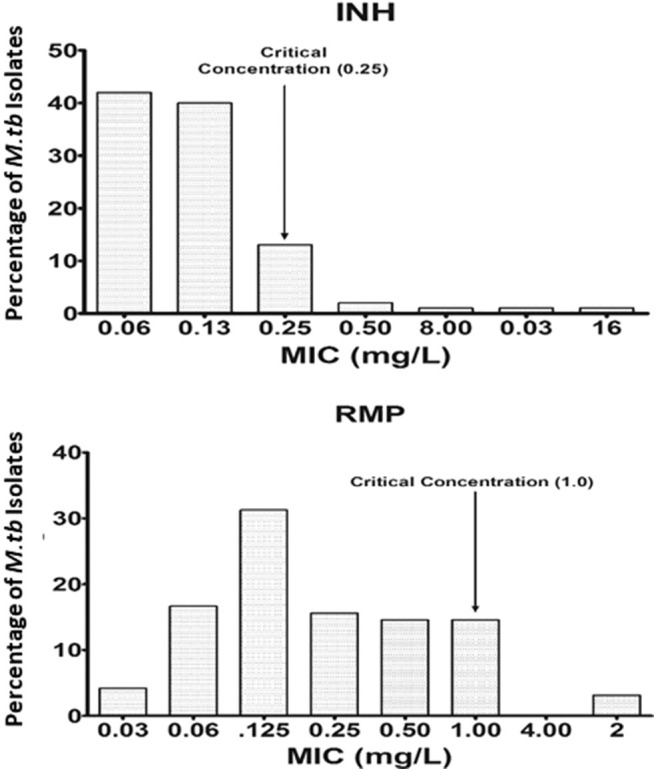
Wild-type minimal inhibitory concentration (MIC) distribution of isoniazid (INH) and rifampicin (RMP) in 96 isolates of *M. tuberculosis* performed in MGIT 960 (liquid media). The critical concentration determined in this study is indicated by the arrow and is the same as recommended by WHO for RMP, whereas for INH, it is 0.25 mg/L as against 0.1 mg/L as recommended by WHO. *Y*-axis represents the percentage of *M. tuberculosis* isolates, and the *X*-axis represents the MIC of the corresponding drugs.

Wild-type MIC distribution of INH and RMP was determined using 276 isolates of *M. tuberculosis*, from the sputum specimens of TB patients before the start of the treatment, in the Middlebrook 7H11 agar plate. The required sample size was not achieved due to the contamination, and it was <4%; hence, there is a slight decrease in the number of tested isolates for the 7H11 agar method. Similarly, it was carried for the same drugs in 96 isolates out of 276 using the MGIT 960 system (liquid medium). The results are shown in Figures 1, 2. The CC was determined using the maximum MIC level of 95% percentage, i.e., 95% percentile of wild type in this study as per the definition is indicated in the respective figures, and this clearly distinguished the susceptible isolates from the resistant ones in the MIC distribution. It was 0.25 and 1.0 mg/L using the 7H11 agar method and the same for MGIT 960 for INH and RMP, respectively. The values of CC recommended by WHO for INH and RMP were 0.2 and 1.0 mg/L for 7H11 agar, and 0.1 and 1.0 mg/L, respectively, for MGIT 960.

Using the observed *C*_max_ and AUC levels in the same population of patients (Hemanth Kumar et al., 2016), the PD was calculated using the CC determined for INH and RMP (Table 1). The optimal level of the PD indices is expected to be between 10 and 100 for the ratios *C*_max_/MIC and AUC/MIC, respectively (Hemanth Kumar et al., 2016). The observed PD level for RMP is found to be below the expected level (5.0 and 27.9; Table 1), but for INH, it is well above the required ratios (45.2 and 164.4; Table 1) for *C*_max_/MIC and AUC/MIC, respectively. Moreover, for eight of the patients, the individualized PD indices were determined with the MICs of the infecting *M. tuberculosis* isolate and their serum levels (*C*_max_ and AUC) in Table 2.

**Table 1:** Results of pharmacokinetics (PK) and pharmacodynamics (PD) for isoniazid (INH) and rifampicin (RMP) obtained in this study.

**Indices**	**RMP**	**INH**
*C*_max_ ^*^	5.0 (3.8–6.9)	11.3 (8.2–13.2)
AUC^*^	27.9 (20.1–33.9)	41.1 (33–59.9)
MIC	1.0	0.25
*C*_max_/MIC	5.0	45.2
AUC/MIC	27.9	164.4

* *C_max_ and AUC values were obtained from Hemanth Kumar et al. (2016)*.

**Table 2:** Individualized minimal inhibitory concentration (MIC), pharmacokinetics (PK) (Hemanth Kumar et al., 2016), and pharmacodynamics (PD) results for isoniazid (INH) and rifampicin (RMP) of eight patients in the study.

**Patients**	**MICs in MGIT 960**		**PK (Hemanth Kumar et al.**, **2016****)**				**PD**			
			**RMP**		**INH**		**RMP**		**INH**	
	**RMP**	**INH**	***C*_**max**_**	**AUC**	***C*_**max**_**	**AUC**	***C*_**max**_/MIC**	**AUC/MIC**	***C*_***max***_/MIC**	**AUC/MIC**
1	0.5	0.5	4.02	29.94	8.49	48.2	**8.04**	**43.88**	16.98	96.4
2	0.5	0.25	3.26	18.56	10.30	37.76	**6.52**	**37.12**	41.2	151.04
3	0.06	0.06	6.20	36.74	13.49	62.78	103.33	612.33	224.8	1046.3
4	0.125	NA	6.29	28.67	NA	NA	50.32	229.36	NA	NA
5	1.0	0.125	6.9	79.53	11.59	39.79	**6.9**	**29.53**	92.72	318.32
6	0.5	0.06	4.82	26.73	8.01	27.21	**9.64**	**53.46**	133.5	453.5
7	0.06	0.06	7.16	46.94	8.75	33.58	119.3	782.3	139.16	559.6
8	0.5	0.125	0.5	2.91	7.71	33.98	**1**	**5.82**	61.68	271.84

*Significant values were highlighted for PD-RMP alone*.

**Figure 3:**
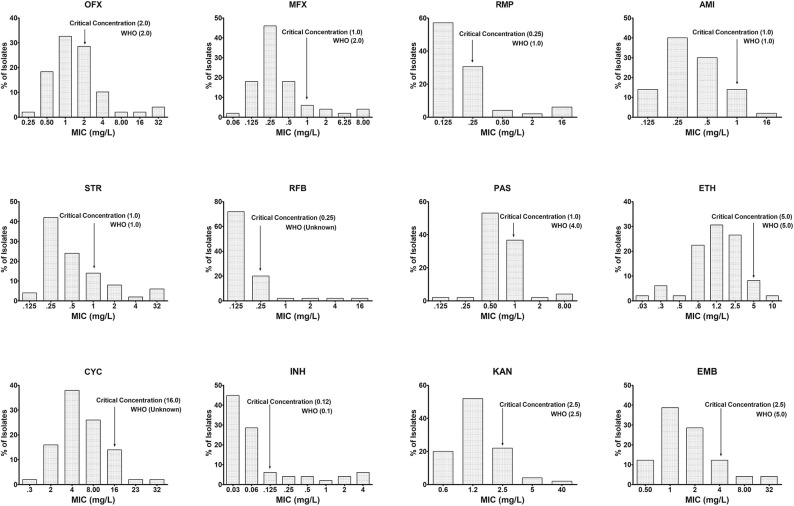
Wild-type minimal inhibitory concentration (MIC) distribution using MYCOTBI plates for first- and second-line anti-TB drugs. The critical concentration determined in this study is indicated by the arrow, and the recommended concentration by WHO for each of the drug could be seen against each of the graph. Deviation from the WHO recommended MIC concentration was observed for RMP, *p*-aminosalicylic acid (PAS), moxifloxacin (MXF), and ethambutol (EMB). *Y*-axis represents the percentage of *M. tuberculosis* isolates, and the *X*-axis represents the MIC of the corresponding drugs.

Similarly, in 50 out of 276 isolates, the MICs of INH, RMP, OFX, MXF, AMI, STR, RFB, PAS, ETM, CYC, KAN, and ETH were determined using the Sensititre MYCOTBI plate. The data are shown in Figure 3. Using this method, we observed that the CC of INH [WHO−0.1 μg/ml; National Institute for Research in Tuberculosis (NIRT)−0.12 μg/ml], RMP (WHO−1 μg/ml; NIRT−0.25 μg/ml), MXF (WHO−2 μg/ml; NIRT−1 μg/ml), para-aminosalicylic acid (WHO−4 μg/ml; NIRT−1 μg/ml), and ETH (WHO−5 μg/ml; NIRT−4 μg/ml) varies from WHO recommendation (Figure 3).

Although both MGIT 960 and MYCOTBI involve the use of 7H9 liquid media, the critical concentration varies depending on the technique used, as seen in RMP and INH. The agreement between the susceptibility results in the 7H11 agar method vs. MGIT and 7H11 vs. MYCOTBI revealed more than 100% agreement with the latter but had 98 and 82.5% for RMP and INH, respectively for the earlier group (Table S1). Concordance and reproducibility were within the acceptable limits when repeatedly tested by each method (Table S2). The difference between the MICs was not more than two dilution steps in all instances, and the susceptibility pattern between 7H11 and MYCOTBI was 100% in comparison with the minimal MIC method using LJ medium and WHO-recommended CC levels for *M. tuberculosis* H37Rv as control. It was repeated thrice for 7H11 and twice for MGIT 960 and MYCOTBI, giving identical results (Table S2). There was a 100% concordance between DST results of 7H11 agar and LJ (minimal MIC method) for interpretation using WHO-recommended CCs for INH and RMP (data not shown). However, there was discordance between MGIT 960 vs. 7H11 agar and LJ among 12 isolates for the RMP susceptibility following WHO recommended CCs. When these 12 isolates were tested using line probe assay (LPA) (GenoType MTBDRplus Version 2.0), they were found to be susceptible to RMP (Table S3).

### *In silico* Analysis of the Whole-Genome Sequence Data

**Figure 4:**
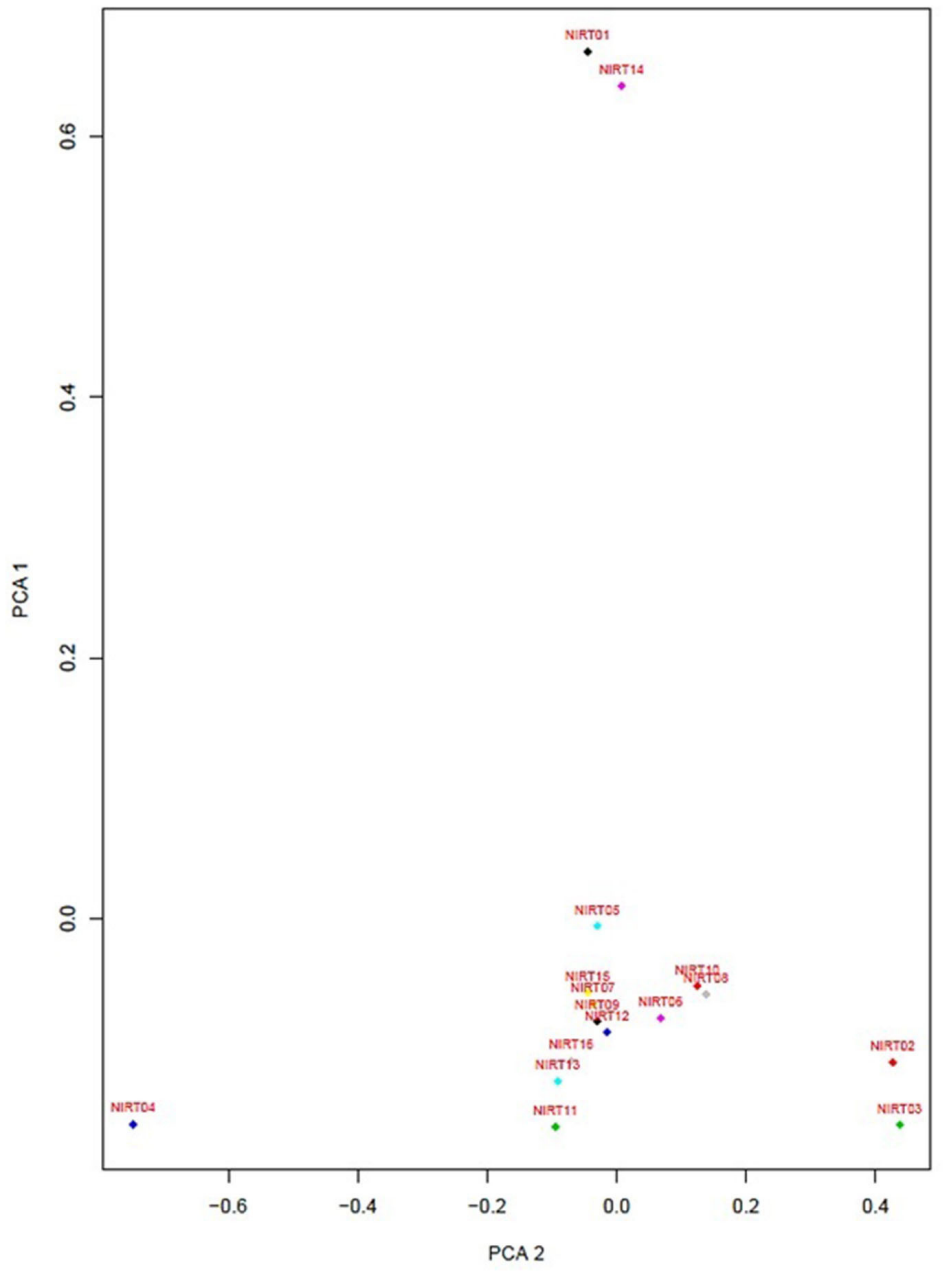
Principle component factor analysis based on the single-nucleotide polymorphisms (SNPs) in the 16 isolates.

**Figure 5:**
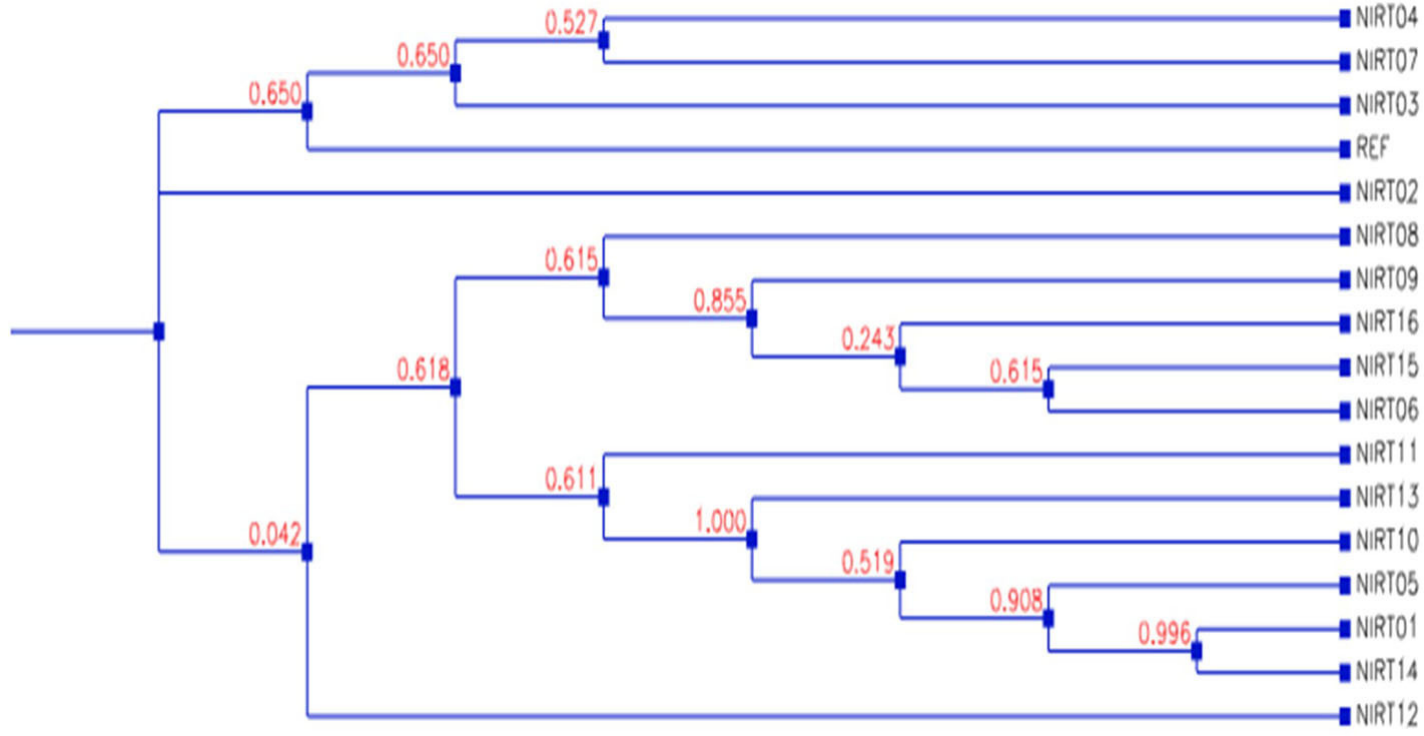
Dendrogram showing the phylogenetic relationship among samples based on genotypes.

From the clinical isolates used in this study, whole-genome sequencing was done for 16 isolates and named as NIRT_01 to NIRT_16. The overall quality of the sequence reads assessed by the FastQC tool was good with a Phred score higher than 30, and there was no adapter content. The primary analysis of the sequences was done (Table S4). In all the sequences, the GC content was ≥65%, thus further establishing the quality of sequencing. The highest number of reads was found in NIRT_14 (47 million reads), and the least was found in NIRT_16 (14 million reads). Using Bowtie2, the filtered quality reads (*Q* > 20) of the 16 clinical samples were mapped against the genome of *M. tuberculosis* H37Rv (accession number: NC_000962.3) considered a reference genome for this study. The overall alignment of these samples resulted in reads, aligned between 95 and 98% against the reference genome with more than 85% of the reads uniquely mapped against the reference. The BAM file after the alignment was subjected to removing PCR duplicate using SAM tools (Table S5). All the samples achieved an overall alignment rate higher than 95% except for two samples. We carried out a phylogenetic analysis to identify the lineage distribution of the 16 samples. The phylogenetic analysis seems to report 13 samples to belong to lineage 1, 2 belong to lineage 2, and 1 sample belongs to lineage 4 (Figure 4, 5 and Table 3). Furthermore, to study the sequence variation among these samples, GATK, Varscan2, and Freebayes2 were used for variant calling. The identified variants that were annotated using SnpEff and SNP Relate (R package) were used for association studies (Tables 4, 5). A total number of 151,010 variants were identified, with the maximum number of variants seen in NIRT_03 and the least were identified in NIRT_14. We removed the common variants present in all isolates that did not account for any phenotype and removed the corresponding genotypes. The resultant SNPs were further annotated for any reported drug resistance activity using drug resistance databases and the literature. Out of the 74 variations found in 16 samples, which could account for the drug resistance activity, 5 of them were identified as novel mutations (Table 6). These five mutations were identified on four different genes such as arabinosyl transferase A (*embA*—Ala217Thr), DNA-directed RNA polymerase subunit beta (*rpoC*—Phe1175Leu), HTH-type transcriptional regulator (*ethR*—Thr130Ile and Ala161Val), and *rrs* (T17G and C517T) genes. Apart from these novel mutations, 24 other known mutations conferring the resistance were also observed (Table 7).

**Table 3:** VCF summary statistics on output VCF files after variant calling and filtering.

**Sequence**	**Seq. type**	**Total variants**	**Transition (Ts)**	**Transversion (Tv)**
NIRT_01	Resistance	13,938	4,506	9,432
NIRT_02	Sensitive	12,396	5,466	6,726
NIRT_03	Sensitive	16,996	6,329	10,667
NIRT_04	Sensitive	12,185	5,173	7,012
NIRT_05	Resistance	9,102	3,488	5,614
NIRT_06	Resistance	10,989	4,521	6,468
NIRT_07	Resistance	14,029	6,251	7,778
NIRT_08	Resistance	13,579	5,364	8,215
NIRT_09	Resistance	6,997	3,440	3,557
NIRT_10	Resistance	7,274	3,291	3,983
NIRT_11	Resistance	5,705	2,818	2,887
NIRT_12	Resistance	6,687	3,186	3,501
NIRT_13	Resistance	5,597	2,797	2,800
NIRT_14	Resistance	4,062	2,025	2,037
NIRT_15	Resistance	5,666	2,453	3,213
NIRT_16	Resistance	5,808	2,250	3,558

**Table 4:** Phenotypic resistance pattern, its corresponding MICs determined by MYCOTBI in parentheses, and genotypic mutated genes.

**Lab No**.	**Phenotypical resistance (MIC)**	**Corresponding genotypical mutated genes**
NIRT_1	INH (0.5)	fabG1, katG
NIRT_4	OFX (16), ETH (20)	gyrA, gyrB
NIRT_5	RMP (2), INH (0.5), EMB (8)	rpoB, rpoC, katG, kasA, embC
NIRT_6	RMP (16), STR (8), PAS (5), RFB (16), CYC (128), INH (4), KAN (5), EMB (8)	rpoB, rpoC, katG, kasA, embC, embA, embB, embR
NIRT_7	OFX (16), INH (4)	gyrA, gyrB, katG, kasA
NIRT_8	INH (0.5)	katG, kasA
NIRT_9	RMP (2), CYC (32), INH (0.25)	rpoB, rpoC, katG, kasA
NIRT_11	OFX (4), INH (4), EMB (8)	gyrA, gyrB, katG, kasA, embC, embA, embB, embR
NIRT_13	INH (0.5)	katG, kasA
NIRT_14	OFX (8), MXF (4), RMP (>16), STR (>32), RFB (4), ETH (10), CYC (32), INH (>4), KAN (16)	gyrA, gyrB, rpoB, rpoC, rpsL, fabG1, KatG
NIRT_15	INH (4, 2)	katG, kasA

**Table 5:** Lineage and strain type identified from the observed genotypes in samples.

**Sample**	**Lineage**	**Name**
NIRT10	Lineage 1	Indo-Oceanic EAI
NIRT06	Lineage 1	Indo-Oceanic EAI
NIRT09	Lineage 1	Indo-Oceanic EAI
NIRT02	Lineage 1	Indo-Oceanic EAI
NIRT03	Lineage 1	Indo-Oceanic EAI
NIRT04	Lineage 1	Indo-Oceanic EAI
NIRT07	Lineage 1	Indo-Oceanic EAI
NIRT08	Lineage 1	Indo-Oceanic EAI
NIRT01	Lineage 2/Beijing sublineage	East-Asian Beijing
NIRT05	Lineage 4/LAM	Euro-American LAM
NIRT14	Lineage 2/Beijing sublineage	East-Asian Beijing
NIRT15	Lineage 1	Indo-Oceanic EAI
NIRT12	Lineage 1	Indo-Oceanic EAI
NIRT11	Lineage 1	Indo-Oceanic EAI
NIRT16	Lineage 1	Indo-Oceanic EAI
NIRT13	Lineage 1	Indo-Oceanic EAI

**Table 6:** Novel potential drug-resistant variants observed in the study.

**S. No**	**Gene name**	**Mutation**
1	*embA*	Ala217Thr
2	*rpoC*	Phe1175Leu
3	*ethR*	Thr130Ile
4	*ethR*	Ala161Val
5	*rrs*	T17G

**Table 7:** Known and previously reported drug-resistant variants observed in the study.

**S. No**	**Gene name**	**Mutation**
1	gyr A	Gly247Ser
2	gyr A	Ala90Val
3	gyr A	Asp94Asn
4	rpoB	His445Arg
5	rpoB	Leu430Pro
6	embC	Ala774Ser
7	embC	Ala387Val
8	embB	Asp354Ala
9	embB	Gln497Lys
10	embB	Val135Met
11	embB	Gly836Arg
12	embB	Met306Ile
13	embB	Phe285Leu
14	embB	Gln497Arg
15	rpoC	Glu750Asp
16	rpoC	Phe831Leu
17	rpoC	Ile491Val
18	rpoC	Ala621Thr
19	kasA	Gly269Ser
20	kasA	Gly312Ser
21	kasA	Phe413Leu
22	ethA	Arg469Trp
23	ethA	Asn345Lys
24	pncA	Gly132Ala

## Discussion

The wild-type MIC distribution of *M. tuberculosis* is necessary for determining the CCs, which in turn are essential for defining its susceptibility as suggested by numerous authors. It necessitates re-evaluation based on the evidence provided regarding the change in the level of certain anti-TB drugs (Schon et al., 2009; Jureen et al., 2010; Werngren et al., 2012). In the current work, as shown in Figures 1, 2, the CC was determined to be identical for RMP in both 7H11 agar and MGIT 960 (1.0 mg/L). However, CC for INH in MGIT 960 was higher than that recommended by WHO. In this study, in a sample size of 96, it was found to be 0.25 mg/L in contrast to 0.1 mg/L as recommended by WHO (Canetti et al., 1963). Since the CCs determined in this study were similar to that prescribed by WHO for RMP using both 7H11 agar and MGIT 960 and in case of solid media alone for INH, the susceptibility pattern was very much similar to that observed using LJ medium by the minimal MIC method for all the isolates used. However, the MICs of 12 isolates were 0.25 mg/L for INH using MGIT 960. They were declared as resistant for INH based on WHO-recommended cutoff, but all these isolates were found to be susceptible to INH using LJ based resistant ratio method and 7H11 agar plates as well.

The lack of clarity in predicting clinical failure in the CC accuracy has led to the establishment of PK and antimicrobial PK/PD developed to detect susceptibility breakpoints for many antibiotics (Dalhoff et al., 2009). However, the outset of Gumbo Monte Carlo simulations defines the new susceptibility breakpoints considering microbiological and clinical consequence and states further that the CCs of first-line drugs could be factually incorrect (Gumbo, 2010). Based on the reason mentioned above and findings, it is necessary to assess the CC of first-line drugs for variability prevailing genotype and polymorphism in a specific area.

Moreover, there were no detectable mutations in the reported genes responsible for drug resistance in INH using LPA (GenoType MTBDRplus Version 2.0) in these 12 isolates. Based on the intention laid by the WHO to reduce the time-consuming culture identification methods and drug resistance findings, the LPA was formulated (World Health Organization, 2007). In the established CC definition, the observed CC would be 0.25 mg/L using wild-type distribution for INH using MGIT 960 witnessed in this study as against WHO's recommended CC. Using this as a baseline value, these 12 isolates can be declared as susceptible, which are similar to the observed DST results based on LJ.

The principles of Canetti et al. (1963), recognized 50 years ago, state to implement a sensitivity test for the capability of organisms growing on a medium containing a wide range of known drugs. Our evaluation of the pivot predominant population was done by MYCOTBI; likewise, the most extensive collection of *M. tuberculosis* isolates from Uganda and the Republic of Korea, at DST level, were assessed by MYCOTBI plate (Lee et al., 2014). Considering the MYCOTBI results in a sample size of 50, changes in the CC were seen in RMP, MXF, ETH, and PAS.

PCA was performed to identify the conformational differences between the resistance patterns (Jamshidi et al., 2018). It classifies the drugs into three groups with data source MIC values of 16 isolates determined by MYCOTBI method, where the EMB, RFB, CYC, and PAS are having a higher variation, while the pattern is followed by other two group drugs as well (Figure S1). PCA, with the data source DST of these 16 isolates, classifies the drugs into two groups; where RMP, MXF, STR, KAN, AMI, OFX, and RFB have a higher variation with the pattern followed by the other two groups of drugs (Figure S2).

Whole-genome sequencing has emerged as a new diagnostic tool that assists in clinical decision making in infectious diseases (Jabbar et al., 2019). The whole-genome sequencing of tuberculosis isolates provides genomic information that is responsible for causing resistance (Cohen et al., 2019). To determine if any correlation exists between high-level resistance and the number of mutations present in the genome of isolates, whole-genome sequencing was done for 16 isolates whose MICs are known (Joshi et al., 2014; Steiner et al., 2014; Coll et al., 2015; Feuerriegel et al., 2015). The mutations related to resistant genes were analyzed, and no correlation between the amount of mutation and the level of resistance was observed. Out of the 74 variants associated with resistance, five were predicted to be new mutations and thus not reported earlier. The novelty and the potential of these five mutations to confer resistance should be evaluated further. Out of the 16 isolates sequenced, NIRT_2, and NIRT_3 were phenotypically sensitive, but had mutations corresponding to fluoroquinolones and ETH. Similarly, discrepancies between genotypic and phenotypic resistance have been reported in ETH, MXF (Chen et al., 2019), and STR (Chaidir et al., 2019). The drug resistance mechanisms are not well-studied (Gygli et al., 2019) especially for second-line anti-TB drugs, and thus, the specificity of the reported genetic mutations to confer phenotypic resistance is low. Apart from genetic mutations, there are also other mechanisms like efflux pumps that confer phenotypic resistance (Satta et al., 2017). Although whole-genome sequencing techniques and its interpretation have become highly advanced, it cannot be used as a standalone tool for predicting the clinical outcome of drug resistance in the current scenario.

Furthermore, innovative approaches need to be adapted to eliminate TB. By implying multidisciplinary, multisectoral approaches, the new Global Tuberculosis Network (GTN) intends to research the much needed therapeutic and diagnostic requirements in the surge to eliminate TB upon applying PK. GTN indeed perceives and studies the existing knowledge slits and renders potential solutions as well (Alffenaar et al., 2019). Thus, our study will be the first to examine the PD implication toward the primary drug line CC of wild-type MIC on *M. tuberculosis* clinical isolates from South India.

Upon analysis based on PD levels in eight patients, the RMP levels were subtherapeutic in five out of eight patients. In 2017, Ramachandran et al. reported suboptimal concentrations of RMP, INH, and pyrazinamide among 91, 16, and 17% of the patients tested. The current study was conducted using the isolates from patients included in the study by Ramachandran et al. (2017).

Using the average serum levels of RMP and INH from this study and the observed MICs of these drugs in the current study using MGIT, PD indices such as *C*_max_/MIC and AUC/MIC ratios were suboptimal for RMP (5.0 and 27.9) and were above the expected limit for INH (45.2 and 164.4). This indicates that these patients received a dose of RMP, which was subtherapeutic in eliminating the pathogen with the prescribed dose for their body weight or else that their physiological system is not capable of maintaining the optimal level of RMP in the serum. In other words, in the majority of these patients, the expected level of action for RMP is suboptimal, and if it had been at the optimal levels, the rate of bacillary eradication and the sputum conversion would be much more rapid.

Although the smaller sample size might be the limitation of the study and a foregone conclusion cannot be arrived based on this sample size, the unexpected result of the subtherapeutic levels found in the majority of these patients to RMP should be noticed and taken care. RMP is one of the essential anti-TB drugs in the regimen to treat tuberculosis, and it implicates the prominence of such a study in a larger sample size, while this well-worn finding will pave the way for an amicable target proposed in terms of End TB Strategy by World Health Organization (2018).

## Conclusion

MICs of anti-TB drugs for the wild-type isolates of *M. tuberculosis* were determined. We were able to predict the CC in both solid and liquid media. The WHO recommended concentration was used to compare the observed CC among the isolates for INH and RMP and was found to be similar in the case of solid media (7H11 agar). Changes in the CCs were noted for INH in MGIT 960, and similar changes were found for MXF, ETH, and PAS in liquid media using MYCOTBI as against the WHO recommended. CC for RMP was found to be identical to that recommended by WHO in both 7H11 and MGIT 960 but differ in MYCOTBI. The therapeutic levels for RMP noted in eight patients were suboptimal for five out of eight patients, and these findings necessitate carrying out a similar exercise in a more significant number of isolates and patients to ascertain the results in this study. The genomes of 16 mycobacterial isolates (including 2 wild types and 14 resistant isolates) were subjected to whole-genome sequencing to identify mutations that can be associated with drug resistance. Two of them were in Beijing, one was Euro-American, and the rests were East African–Indian (EAI) strain types. A total of 74 mutations were identified that are known to be associated with drug resistance in these isolates, while five mutations were identified that appeared to be novel and required further analysis to confer their role in resistance. These findings highlight the need for optimizing the drug dosage based on PD from large-scale studies in different geographical settings.

## Data Availability Statement

The gene sequence from the 16 isolates has been submitted to the NCBI repository with SRA accession: PRJNA596377 (https://www.ncbi.nlm.nih.gov/bioproject/PRJNA596377).

## Ethics Statement

The studies involving human participants were reviewed and approved by Institutional Ethics Committee of National Institute for Research in Tuberculosis, Chennai, India. The patients/participants provided their written informed consent to participate in this study.

## Author Contributions

AD conceptualized the study. AD, SS, MB, and AB performed the bacteriological part of the study. SH and MT performed the analysis of the whole-genome sequence. KT did the statistical analysis for this study. GR and AK were involved in the PK part of the study. The first draft of the manuscript was written by AD and SS. AZ, CN, SR, SM, and RM oversaw the final analysis and writing of this manuscript. All authors contributed to the article and approved the submitted version.

## Conflict of Interest

The authors declare that the research was conducted in the absence of any commercial or financial relationships that could be construed as a potential conflict of interest.
