# Supplementary material for: Wild-Type MIC Distribution for Re-evaluating the Critical Concentration of Anti-TB Drugs and Pharmacodynamics Among Tuberculosis Patients From South India
Source: Front Microbiol. 2020 Jun 30;11:1182. doi: 10.3389/fmicb.2020.01182 (PMC7338667; doi:10.3389/fmicb.2020.01182)

**SUPPLEMENTARY MATERIAL**

**TABLE S1.**Agreement for RMP and INH between 7H11, MYCOTBI and MGIT960

| **RMP** | | | | |  | **INH** | | | | |
| --- | --- | --- | --- | --- | --- | --- | --- | --- | --- | --- |
| **7H11** |  | **MYCOTBI** | | |  | **7H11** |  | **MYCOTBI** | | |
|  |  | **Res.** | **Sens.** | **Total** |  |  |  | **Res.** | **Sens.** | **Total** |
|  | **Res.** | 1 | 0 | 1 |  |  | **Res.** | 6 | 0 | 6 |
|  |  | 100.0% | 0.0% |  |  |  |  | 100.0% | 0.0% |  |
|  | **Sens.** | 0 | 36 | 36 |  |  | **Sens.** | 0 | 31 | 31 |
|  |  | 0.0% | 100.0% |  |  |  |  | 0.0% | 100.0% |  |
|  | **Total** | **1** | **36** | **37** |  |  | **Total** | **6** | **31** | **37** |
|  | **Kappa** | 100% | | |  |  | **Kappa** | 100% | | |
|  | **Sensitivity** | 100% (25% - 100%) | | |  |  | **Sensitivity** | 100% (54.1% - 100%) | | |
|  | **Specificity** | 100% (90.3% - 100%) | | |  |  | **Specificity** | 100% (88.8% - 100%) | | |
|  | **PPV** | 100% | | |  |  | **PPV** | 100% | | |
|  | **NPV** | 100% | | |  |  | **NPV** | 100% | | |
|  | **Accuracy** | 100% (90.5% - 100%) | | |  |  | **Accuracy** | 100% (90.5% - 100%) | | |
| **MGIT** |  | **MYCOTBI** | | |  | **MGIT** |  | **MYCOTBI** | | |
|  |  | **Res.** | **Sens.** | **Total** |  |  |  | **Res.** | **Sens.** | **Total** |
|  | **Res.** | 0 | 0 | 0 |  |  | **Res.** | 1 | 3 | 4 |
|  |  |  | 0.0% |  |  |  |  | 100.0% | 50.0% |  |
|  | **Sens.** | 0 | 6 | 6 |  |  | **Sens.** | 0 | 3 | 3 |
|  |  |  | 100.0% |  |  |  |  | 0.0% | 50.0% |  |
|  | **Total** | **0** | **6** | **6** |  |  | **Total** | **1** | **6** | **7** |
|  | **Kappa** | NA | | |  |  | **Kappa** | 57.1% | | |
|  | **Sensitivity** | NA | | |  |  | **Sensitivity** | 100% (2.5% - 100%) | | |
|  | **Specificity** | 100% (54.1% - 100%) | | |  |  | **Specificity** | 50.0% (11.8% - 88.9%) | | |
|  | **PPV** | NA | | |  |  | **PPV** | 25.0% (13.0% - 42.6%) | | |
|  | **NPV** | 100% | | |  |  | **NPV** | 100% | | |
|  | **Accuracy** |  | | |  |  | **Accuracy** | 57.1% (18.4% - 90.1%) | | |
| **7H11** |  | **MGIT** | | |  | **7H11** |  | **MGIT** | | |
|  |  | **Res.** | **Sens.** | **Total** |  |  |  | **Res.** | **Sens.** | **Total** |
|  | **Res.** | 2 | 0 | 2 |  |  | **Res.** | 2 | 0 | 2 |
|  |  | 50.0% | 0.0% |  |  |  |  | 10.5% | 0.0% |  |
|  | **Sens.** | 2 | 96 | 98 |  |  | **Sens.** | 17 | 78 | 95 |
|  |  | 50.0% | 100.0% |  |  |  |  | 89.5% | 100.0% |  |
|  | **Total** | **4** | **96** | **100** |  |  | **Total** | **19** | **78** | **97** |
|  | **Kappa** | 98.0% | | |  |  | **Kappa** | 82.5% | | |
|  | **Sensitivity** | 50.0% (6.8% - 93.2%) | | |  |  | **Sensitivity** | 10.5% (1.3% - 33.1%) | | |
|  | **Specificity** | 100% (96.2% - 100%) | | |  |  | **Specificity** | 100% (95.4% - 100%) | | |
|  | **PPV** | 100% | | |  |  | **PPV** | 100% | | |
|  | **NPV** | 98.0% (94.7% - 99.2%) | | |  |  | **NPV** | 82.1% (79.7% - 84.3%) | | |
|  | **Accuracy** | 98.0% (93.0% - 99.8%) | | |  |  | **Accuracy** | 82.5% (73.4% - 89.5%) | | |

**TABLE S2:** Reproducibility of the results among replicates

‘C’ denotes Contamination

|  |  |  |  |  |  | **MYCOTBI** | | | | | | | | | | | |
| --- | --- | --- | --- | --- | --- | --- | --- | --- | --- | --- | --- | --- | --- | --- | --- | --- | --- |
| **LAB NO** | **REPLICATES** | **MEDIA** | **RMP** | **INH** |  | **OFX** | **MXF** | **RMP** | **AMI** | **STR** | **RFB** | **PAS** | **ETH** | **CYC** | **INH** | **KAN** | **EMB** |
| 1193 | 1 | 7H11 | 0.5 | 0.125 |  | 2 | 0.5 | <0.12 | 1 | 0.5 | <0.12 | <0.5 | 2.5 | 16 | 0.06 | 1.2 | 2 |
| 1193 | 2 | 7H11 | 0.5 | 0.125 |  |  |  |  |  |  |  |  |  |  |  |  |  |
| 512 | 1 | 7H11 | 0.125 | 0.125 |  | 1 | 0.25 | <0.12 | 0.25 | <0.25 | <0.12 | 1 | 1.2 | <2 | <0.03 | 1.2 | 1 |
| 512 | 2 | 7H11 | 0.125 | 0.25 |  |  |  |  |  |  |  |  |  |  |  |  |  |
| 72 | 1 | 7H11 | 0.125 | 0.25 |  |  |  |  |  |  |  |  |  |  |  |  |  |
| 72 | 2 | 7H11 | 0.25 | 0.125 |  |  |  |  |  |  |  |  |  |  |  |  |  |
| 350 | 1 | 7H11 | 0.125 | 0.125 |  |  |  |  |  |  |  |  |  |  |  |  |  |
| 350 | 2 | 7H11 | 0.25 | 0.125 |  |  |  |  |  |  |  |  |  |  |  |  |  |
| H37Rv | 1 | 7H11 | 0.25 | 0.06 |  | 0.5 | 0.12 | <0.12 | <0.12 | <0.25 | <0.12 | 4 | 0.6 | 32 | 0.06 | 1.2 | 2 |
| H37Rv | 2 | 7H11 | 0.25 | 0.06 |  | 0.5 | 0.12 | <0.12 | <0.12 | <0.25 | <0.12 | 4 | 0.6 | 16 | 0.06 | 1.2 | 1 |
| H37Rv | 3 | 7H11 | 0.25 | 0.06 |  |  |  |  |  |  |  |  |  |  |  |  |  |
| 96 |  | 7H11 | 0.25 | NA |  | 0.5 | 0.12 | <0.12 | <0.12 | <.25 | <0.12 | <0.5 | 0.6 | 4 | 0.06 | 1.2 | 1 |
| 102 |  | 7H11 | 0.25 | 0.125 |  | 1 | 0.12 | <0.12 | 0.25 | <0.25 | <0.12 | <0.5 | 1.2 | 4 | <0.03 | 1.2 | 0.5 |
| 1267 |  | 7H11 | 0.25 | 8 |  | 1,2 | C | <0.12 | 0.25 | 1 | <0.12 | <0.5 | 2.5 | 4 | 4,2 | 1.2 | 1 |
| 1077 |  | 7H11 | 0.5 | 0.25 |  | 0.5 | 0.25 | <0.12 | 0.5 | <0.25 | <0.12 | C | C | 4 | C | 1.2 | C |
| H37Rv |  | MGIT | 0.2 | <0.03 |  |  |  |  |  |  |  |  |  |  |  |  |  |
| H37Rv |  | MGIT | 0.2 | <0.03 |  |  |  |  |  |  |  |  |  |  |  |  |  |
| 238 | 1 | MGIT | 0.25 | 0.25 |  |  |  |  |  |  |  |  |  |  |  |  |  |
| 238 | 2 | MGIT | 0.25 | NA |  |  |  |  |  |  |  |  |  |  |  |  |  |
| 64 | 1 | MGIT | 0.25 | 0.25 |  |  |  |  |  |  |  |  |  |  |  |  |  |
| 64 | 2 | MGIT | 0.5 | 0.5 |  |  |  |  |  |  |  |  |  |  |  |  |  |
| 349 | 1 | MGIT | >0.5 | 0.25 |  |  |  |  |  |  |  |  |  |  |  |  |  |
| 349 | 2 | MGIT | 0.5 | 0.25 |  |  |  |  |  |  |  |  |  |  |  |  |  |
| 565 | 1 | MGIT | 0.25 | 0.25 |  |  |  |  |  |  |  |  |  |  |  |  |  |
| 565 | 2 | MGIT | 0.25 | NA |  |  |  |  |  |  |  |  |  |  |  |  |  |
| 1171 | 1 | MGIT | 1 | 0.5 |  |  |  |  |  |  |  |  |  |  |  |  |  |
| 1171 | 2 | MGIT | NA | 0.25 |  |  |  |  |  |  |  |  |  |  |  |  |  |
| 878 | 1 | MGIT | 1 | 0.125 |  |  |  |  |  |  |  |  |  |  |  |  |  |
| 878 | 2 | MGIT | NA | 0.25 |  |  |  |  |  |  |  |  |  |  |  |  |  |
| 648 | 1 | MGIT | 1 | 0.25 |  |  |  |  |  |  |  |  |  |  |  |  |  |
| 648 | 2 | MGIT | NA | 0.5 |  |  |  |  |  |  |  |  |  |  |  |  |  |
| 1128 | 1 | MGIT | 1 | 0.5 |  |  |  |  |  |  |  |  |  |  |  |  |  |
| 1128 | 2 | MGIT | NA | 0.5 |  |  |  |  |  |  |  |  |  |  |  |  |  |
| 1190 | 1 | MGIT | 0.5 | 0.5 |  |  |  |  |  |  |  |  |  |  |  |  |  |
| 1190 | 2 | MGIT | NA | 0.5 |  |  |  |  |  |  |  |  |  |  |  |  |  |
| 1205 | 1 | MGIT | 0.5 | 0.25 |  |  |  |  |  |  |  |  |  |  |  |  |  |
| 1205 | 2 | MGIT | NA | 0.25 |  |  |  |  |  |  |  |  |  |  |  |  |  |
| 224 | 1 | MGIT | 0.125 | 0.25 |  |  |  |  |  |  |  |  |  |  |  |  |  |
| 224 | 2 | MGIT | NA | 0.25 |  |  |  |  |  |  |  |  |  |  |  |  |  |
| 288 | 1 | MGIT | 0.25 | >1 |  |  |  |  |  |  |  |  |  |  |  |  |  |
| 288 | 2 | MGIT | NA | 2 |  |  |  |  |  |  |  |  |  |  |  |  |  |
| 1847 | 1 | MGIT | 0.5 | 0.25 |  |  |  |  |  |  |  |  |  |  |  |  |  |
| 1847 | 2 | MGIT | 0.5 | 0.25 |  |  |  |  |  |  |  |  |  |  |  |  |  |
| 1914 | 1 | MGIT | 0.125 | 0.25 |  |  |  |  |  |  |  |  |  |  |  |  |  |
| 1914 | 2 | MGIT | NA | 0.25 |  |  |  |  |  |  |  |  |  |  |  |  |  |

|  |  |  |  |  |  |  |  |  |  |
| --- | --- | --- | --- | --- | --- | --- | --- | --- | --- |

**TABLE S3:** LPA results of 12 isolates in comparison with 7H11 agar method and MGIT960

| **Lab no** | **Replicates** | **Method** | **RMP** | **INH** |  | **TUB** | **rpoB WT** | **rpoB MUT** | **katG WT** | **katG MUT** | **inhA WT** | **inhA MUT** | **RMP** | **INH** |
| --- | --- | --- | --- | --- | --- | --- | --- | --- | --- | --- | --- | --- | --- | --- |
| 238 |  | 7H11 | 0.125 | 0.125 |  | P | P | N | P | N | P | N | S | S |
| 238 |  | MGIT | 0.25 | 0.25 |  |  |  |  |  |  |  |  |  |  |
| 64 |  | 7H11 | 0.125 | 0.125 |  | P | P | N | P | N | P | N | S | S |
| 64 | 1 | MGIT | 0.25 | 0.25 |  |  |  |  |  |  |  |  |  |  |
| 64 | 2 | MGIT | 0.5 | 0.5 |  |  |  |  |  |  |  |  |  |  |
| 349 |  | 7H11 | 0.5 | 0.06 |  | P | P | N | P | N | P | N | S | S |
| 349 | 1 | MGIT | >0.5 | 0.25 |  |  |  |  |  |  |  |  |  |  |
| 349 | 2 | MGIT | 0.5 | 0.25 |  |  |  |  |  |  |  |  |  |  |
| 565 |  | 7H11 | 0.125 | 0.125 |  | P | P | N | P | N | P | N | S | S |
| 565 |  | MGIT | 0.25 | 0.25 |  |  |  |  |  |  |  |  |  |  |
| 775 |  | 7H11 | 0.125 | 0.06 |  | P | P | N | P | N | P | N | S | S |
| 775 |  | MGIT | 0.25 | 0.25 |  |  |  |  |  |  |  |  |  |  |
| 775 |  | MGIT | 0.25 | 0.25 |  |  |  |  |  |  |  |  |  |  |
| 1171 |  | 7H11 | 0.5 | 0.25 |  | P | P | N | P | N | P | N | S | S |
| 1171 | 1 | MGIT | 1 | 0.5 |  |  |  |  |  |  |  |  |  |  |
| 1171 | 2 | MGIT |  | 0.25 |  |  |  |  |  |  |  |  |  |  |
| 648 |  | 7H11 | 1 | 0.25 |  | P | P | N | P | N | P | N | S | S |
| 648 | 1 | MGIT | 1 | 0.25 |  |  |  |  |  |  |  |  |  |  |
| 648 | 2 | MGIT | NA | 0.5 |  |  |  |  |  |  |  |  |  |  |
| 1205 |  | 7H11 | 0.25 | 0.125 |  | P | P | N | P | N | P | N | S | S |
| 1205 | 1 | MGIT | 0.5 | 0.25 |  |  |  |  |  |  |  |  |  |  |
| 1205 | 2 | MGIT | NA | 0.25 |  |  |  |  |  |  |  |  |  |  |
| 224 (225) |  | 7H11 | 0.06 | 0.125 |  | P | P | N | P | N | P | N | S | S |
| 224 (225) | 1 | MGIT | 0.125 | 0.25 |  |  |  |  |  |  |  |  |  |  |
| 224 (225) | 2 | MGIT | NA | 0.25 |  |  |  |  |  |  |  |  |  |  |
| 288 (242) |  | 7H11 | 0.125 | 0.25 |  | P | P | N | P | N | P | N | S | S |
| 288 (242) | 1 | MGIT | 0.25 | >1 |  |  |  |  |  |  |  |  |  |  |
| 288 (242) | 2 | MGIT | NA | 2 |  |  |  |  |  |  |  |  |  |  |
| 1847 |  | 7H11 | 0.25 | 0.25 |  | P | P | N | P | N | P | N | S | S |
| 1847 | 1 | MGIT | 0.5 | 0.25 |  |  |  |  |  |  |  |  |  |  |
| 1847 | 2 | MGIT | 0.5 | 0.25 |  |  |  |  |  |  |  |  |  |  |
| 1914 |  | 7H11 | 0.125 | 0.125 |  | P | P | N | P | N | P | N | S | S |
| 1914 | 1 | MGIT | 0.125 | 0.25 |  |  |  |  |  |  |  |  |  |  |
| 1914 | 2 | MGIT | NA | 0.25 |  |  |  |  |  |  |  |  |  |  |

**P-Positive**

**N-Negative**

**S- Sensitive**

**TABLE S4:** Quality assessment information on sequencing reads before preprocessing.

| **Sequence** | **Total Sequences** | **%GC** |
| --- | --- | --- |
| NIRT_01 | 2741211 | 65/66 |
| NIRT_02 | 3163434 | 65 |
| NIRT_03 | 2873926 | 65 |
| NIRT_04 | 3060696 | 66 |
| NIRT_05 | 2907517 | 61 |
| NIRT_06 | 3307710 | 64 |
| NIRT_07 | 3089637 | 65 |
| NIRT_08 | 3897740 | 65 |
| NIRT_09 | 4175858 | 65 |
| NIRT_10 | 3603052 | 66 |
| NIRT_11 | 4350729 | 65 |
| NIRT_12 | 4428489 | 65 |
| NIRT_13 | 4227343 | 65 |
| NIRT_14 | 4741164 | 66 |
| NIRT_15 | 1525242 | 65 |
| NIRT_16 | 1423445 | 65 |

**TABLE S5:** Alignment statistics on sequence reads after aligning with reference genome

| **Sequence** | **Aligned exactly 1 time (%)** | **Aligned more than 1 time (%)** | **Aligned 0 time**  **(%)** | **Overall alignment rate (%)** |
| --- | --- | --- | --- | --- |
| NIRT_01 | 88.49 | 2.73 | 8.76 | 98.74 |
| NIRT_02 | 86.01 | 2.21 | 11.87 | 95.78 |
| NIRT_03 | 90.72 | 2.30 | 6.98 | 97.24 |
| NIRT_04 | 88.33 | 2.21 | 9.46 | 98.29 |
| NIRT_05 | 73.63 | 1.99 | 24.37 | 82.03 |
| NIRT_06 | 84.49 | 2.04 | 13.47 | 93.64 |
| NIRT_07 | 76.29 | 1.77 | 21.95 | 83.62 |
| NIRT_08 | 87.37 | 2.06 | 10.58 | 98.16 |
| NIRT_09 | 87.36 | 2.00 | 10.63 | 97.54 |
| NIRT_10 | 90.23 | 2.37 | 7.40 | 98.19 |
| NIRT_11 | 87.86 | 2.18 | 9.96 | 98.15 |
| NIRT_12 | 81.75 | 2.31 | 15.94 | 96.55 |
| NIRT_13 | 86.95 | 2.06 | 10.99 | 97.52 |
| NIRT_14 | 85.15 | 2.61 | 12.25 | 97.21 |
| NIRT_15 | 85.72 | 3.07 | 11.21 | 97.56 |
| NIRT_16 | 89.47 | 3.06 | 7.47 | 97.63 |

**FIGURE 1:** Principle component factor analysis with data source of MIC values of 16 isolates determined by MYCOTBI method.


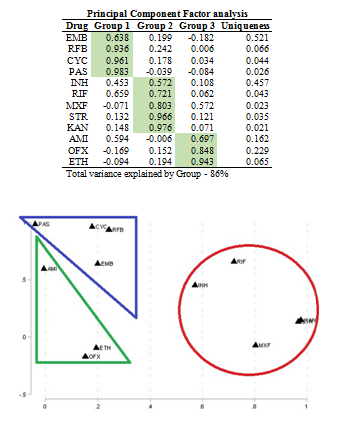


**FIGURE 2:** Principle component factor analysis with data source of sensitive or resistance of 16 isolates determined by MYCOTBI method.


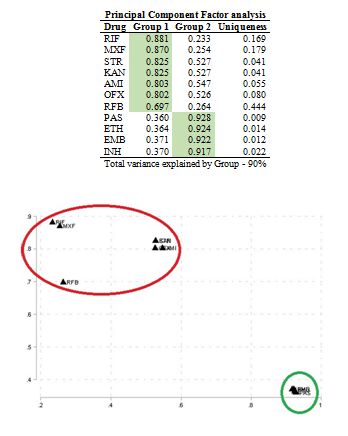

Supplement: Supplementary file 1 [file Data_Sheet_1.docx]
